# A systematic review of the association of Type I diabetes with sensorineural hearing loss

**DOI:** 10.1371/journal.pone.0298457

**Published:** 2024-02-09

**Authors:** Rahul Mittal, Keelin McKenna, Grant Keith, Joana R. N. Lemos, Jeenu Mittal, Khemraj Hirani

**Affiliations:** 1 Diabetes Research Institute, University of Miami Miller School of Medicine, Miami, Florida, United States of America; 2 Department of Otolaryngology, University of Miami Miller School of Medicine, Miami, Florida, United States of America; 3 Herbert Wertheim College of Medicine, Florida International University, Miami, Florida, United States of America; 4 School of Medicine and Public Health, University of Wisconsin, Madison, Wisconsin, United States of America; University of Michigan Medical School, UNITED STATES

## Abstract

**Objectives:**

Type 1 diabetes (T1D) has been associated with several comorbidities such as ocular, renal, and cardiovascular complications. However, the effect of T1D on the auditory system and sensorineural hearing loss (SNHL) is still not clear. The aim of this study was to conduct a systematic review to evaluate whether T1D is associated with hearing impairment.

**Methods:**

The databases PubMed, Science Direct, Scopus, and EMBASE were searched in accordance with the Preferred Reporting Items for Systematic Reviews and Meta-Analyses (PRISMA) criteria. Three reviewers independently screened, selected, and extracted data. The Joanna Briggs Institute (JBI) Critical Appraisal Tools for Analytical cross-sectional and case-control studies were used to perform quality assessment and risk of bias analysis on eligible studies.

**Results:**

After screening a total of 463 studies, 11 eligible original articles were included in the review to analyze the effects of T1D on the auditory system. The included studies comprised cross-sectional and case-control investigations. A total of 5,792 patients were evaluated across the 11 articles included. The majority of the studies showed that T1D was associated with hearing impairment compared to controls, including differences in PTAs and OAEs, increased mean hearing thresholds, altered acoustic reflex thresholds, and problems with the medial olivocochlear (MOC) reflex inhibitory effect. Significant risk factors included older age, increased disease duration, and higher HbA1C levels.

**Conclusions:**

This systematic review suggests that there is a correlation between T1D and impairment on the auditory system. A multidisciplinary collaboration between endocrinologists, otolaryngologists, and audiologists will lead to early detection of hearing impairment in people with T1D resulting in early intervention and better clinical outcomes in pursuit of improving the quality of life of affected individuals.

**Registration:**

This systematic review is registered in PROSPERO (CRD42023438576).

## Introduction

Type 1 diabetes (T1D) is a metabolic disorder that is characterized by autoimmune destruction of the beta (β) cells of the pancreas, resulting in decreased insulin production [1–6]. The autoimmune reaction is primarily propagated by CD4+ and CD8+ cells and macrophages that can enter and destroy the islets [7, 8]. Destruction of pancreatic beta cells ultimately leads to chronic hyperglycemia due to decreased insulin-mediated entry of glucose into cells [9]. Long-standing hyperglycemia triggers microvascular damage, particularly in the retina, heart, and kidneys. This may lead to blindness, retinal neuropathy, cardiovascular complications, and chronic renal disease, which is a significant cause of morbidity and mortality in individuals with diabetes [10–12].

T1D is highly prevalent globally and has been increasing steadily [13–16]. In 2021, it was reported that approximately 8.4 million individuals had a diagnosis of T1D worldwide. Of these individuals, 1.5 million were under the age of 20, 5.4 million were aged 20–59 years, and 1.6 million were over the age of 60 years. Moreover, 1.8 million were in low-income or lower-middle-income countries. This number is predicted to continue to rise with cases reaching an estimated 13.5–17.4 million worldwide by 2040 [17].

In addition to cardiovascular, renal, and ophthalmic complications, it has been hypothesized that chronic hyperglycemia may negatively impact the microvasculature of the inner ear, causing thickening of the capillary walls and, subsequently, decreased blood flow. Although this has not been well studied in humans, it has been shown in animal models that the microangiopathy associated with T1D may lead to damage of the inner ear due to decreased nutrient transport [18]. This process can lead to dysfunction of the sensory structures in the inner ear such as the stria vascularis, organ of Corti, and neurons of the cochlea, leading to irreversible sensorineural hearing loss (SNHL) [19].

**Fig 1:**
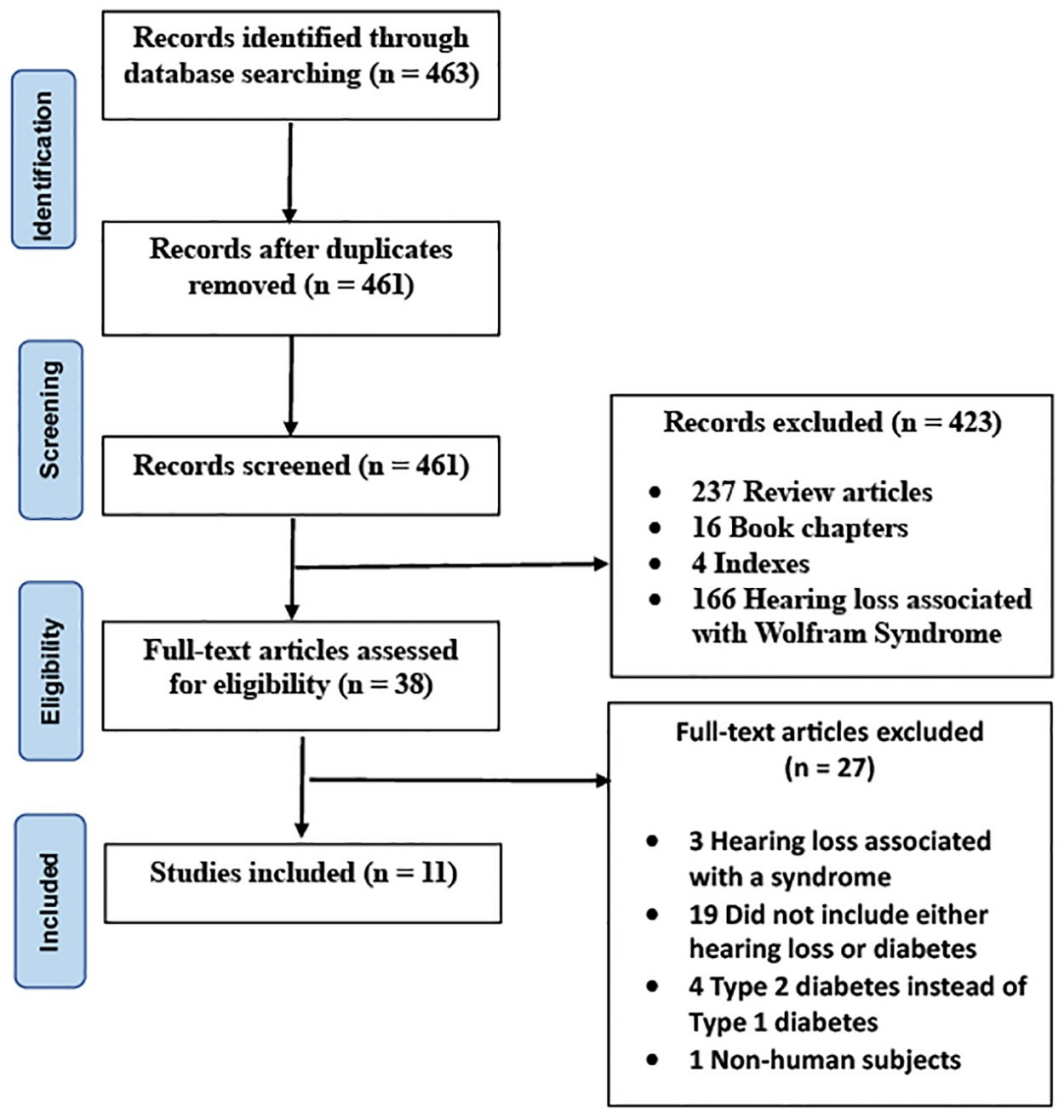
Preferred reporting items for systematic reviews and meta-analyses (PRISMA) flowchart showing the article selection process.

Although the exact prevalence of SNHL in individuals with T1D is unknown, the National Health and Nutrition Examination Survey (NHANES) has shown a twofold greater prevalence of hearing impairment in individuals with diabetes compared to those who do not have the disease [20]. These findings are very concerning as SNHL has been shown to be associated with a significant decline in social and physical functioning as well as quality of life [21–23]. Importantly, it has been demonstrated that SNHL is a significant risk factor for cognitive decline and dementia [24–27]. These factors ultimately lead to a substantial rise in both medical and non-medical costs [28–32]. The objective of this systematic review is to critically appraise the current literature in order to better understand the prevalence of SNHL in individuals with T1D, which is currently greatly underappreciated in people with diabetes. We discuss the impact of T1D on both children and adults as well as identify potential risk factors associated with hearing impairment. To ensure rigor in our study, we have included studies that assessed hearing thresholds using well-established measures such as PTA, DPOAEs, and ABRs as shown in preferred reporting items for systematic reviews and meta-analyses (PRISMA) diagram (Fig 1).

The inner ear itself is a complex structure specializing in both hearing and balance. The cochlea is the inner structure that receives mechanical, auditory vibrations and transduces them into an electrical signal, which is then perceived by the brain leading to the sound perception [33–41]. The cochlea is divided into three spaces: the scala vestibuli, scala media, and scala tympani. These ducts are fluid filled and transmit sound waves that are perceived as different frequencies along the length of the cochlea [33, 34]. The scala media contains an additional three important components: the Reissner’s membrane (RM), which acts as a barrier from the scala vestibuli; the stria vascularis (SV), which generates an ideal electrochemical environment, and the organ of Corti (OC), which is the sensory epithelium [33, 34]. The inner hair cells (IHCs) and outer hair cells (OHCs) on the OC are the main transducers of the mechanical signal. When the pressure waves cause deformation of the hair cells, they depolarize and release neurotransmitters. The hair cells synapse with the bipolar neurons of the spiral ganglion. These nerves project centrally to the cochlear nucleus and stimulate the auditory nerves, which carry the signal to the brain [33, 34].

The auditory function and hearing thresholds can be determined clinically in several ways [42, 43]. The first technique is the auditory evoked potential (AEP), which evaluates neural activity from the cochlea to the auditory cortex [44, 45]. The short-latency AEP is the auditory brainstem response (ABR). ABR determines the electrical response to sounds, when it is converted in electrical impulses from the eighth cranial nerve through the brainstem that is represented as waves I to V in a graphical representation. This is recorded on the scalp of the individual during the first 10ms after the stimulus. This technique is particularly useful in infants as it can be performed without participation from the subject under testing [44]. The second technique is the pure-tone audiometry (PTA) that is used to determine hearing thresholds at different frequencies, usually 125–8000 Hz, which is considered speech frequency [46, 47]. Decibel level is adjusted at each frequency until the lowest decibel required to hear the frequency is identified [48]. The results are then plotted with hertz on the horizontal axis and decibels on the vertical axis. The plots can then be interpreted to assess for conductive hearing loss vs. SNHL [48]. PTA involves both peripheral and central auditory systems. A third technique is recording otoacoustic emissions (OAEs). This can be further subdivided into distortion product otoacoustic emissions (DPOAEs) and transitory evoked otoacoustic emissions (TEOAEs), which provide information about the health of the OHCs [49–51]. When stimulated, the cochlea produces electromechanical feedback from the OHCs, which can be measured in the ear canal. DPOAEs are specific in that they are measured as an intermodulation frequency when the ear is stimulated by two tones [52]. In contrast to DPOAEs, TEOAEs are measured after emitting an acoustic stimulus of a very short duration [51]. These results can be utilized to screen for SNHL and provide information about cochlear mechanics.

This systematic review identified the importance of hearing evaluation in individuals with T1D. Exploring the mechanisms by which hyperglycemia could affect hearing would provide a deeper understanding of the pathophysiology involved. This could include the effects of high blood sugar on blood vessels and nerves in the ear, leading to conditions such as cochlear hydrops or diabetic neuropathy affecting auditory pathways. Addressing the link between hyperglycemia and hearing impairment would have significant clinical implications. It would highlight the importance of blood sugar control in preventing or managing hearing problems in diabetic patients, offering practical insights for healthcare providers and patients.

Studies have established a connection between hyperglycemia and hearing impairment in individuals with T1D [18, 19]. High blood sugar levels can damage the small blood vessels in the body, including those in the inner ear. The inner ear requires a rich blood supply for normal functioning, and damage to these vessels can lead to hearing impairment [53]. Hyperglycemia can lead to increased oxidative stress and inflammation throughout the body [54, 55]. These processes can damage the delicate sensory structures within the ear, such as the hair cells that are crucial for hearing. The cochlea within the inner ear may experience altered metabolic processes due to high blood sugar. This could affect its function and lead to hearing impairment.

In this article, we focused on T1D and not on type 2 diabetes (T2D) as T1D is characterized by the autoimmune destruction of pancreatic beta cells, leading to absolute insulin deficiency [1–6]. This clear and distinct pathophysiology allows for more straightforward study of the direct effects of insulin deficiency and hyperglycemia on auditory function, without the confounding factors present in T2D, such as insulin resistance, and varying degrees of insulin deficiency [56–58], making it difficult to make valid comparisons across different studies. T1D typically is not accompanied with the additional metabolic disorders often associated with T2D, such as obesity, hypertension, and dyslipidemia [59–61]. This simplifies the study of the direct impact of hyperglycemia on SNHL, without the interference of these co-morbidities. In addition, T1D usually has an earlier onset and more rapid progression than T2D [1–6]. This allows researchers to observe the effects of diabetes on hearing over a shorter time frame, which can be particularly useful in longitudinal studies. Furthermore, T1D treatment primarily involves insulin therapy [1, 2], whereas T2D management can include a variety of medications, such as metformin, sulfonylureas, thiazolidinediones, and newer classes like SGLT2 inhibitors and GLP-1 receptor agonists [62–67]. The complexity and variability of T2D medications can introduce confounding factors, making it difficult to isolate the effects of the disease itself on SNHL. T1D offers more consistency in terms of treatment (insulin) across different studies, which is advantageous for comparing and synthesizing research findings. In contrast, the diversity in T2D treatments across different studies could lead to inconsistent results.

Enhancing the understanding of the prevalence of SNHL in individuals with T1D will provide novel avenues for preventing and treating this condition, ultimately improving the quality of life for those already living with this debilitating chronic disease. Once we a get a better understanding of T1D on auditory function, it is possible that insights gained from T1D can be relevant for understanding SNHL in T2D as hyperglycemia is a common factor in both types of diabetes.

## Methods

### Search strategy

This study was carried out in accordance with the guidelines set forth by the Preferred Reporting Items for Systematic Reviews and Meta-Analyses (PRISMA) and was further enhanced by following the recommendations provided in the Cochrane Collaboration Handbook [68]. A protocol of this systematic review was designed *a priori* and subsequently registered in the PROSPERO database prior to the commencement of the study (registration number: CRD42023438576). As narrative or systematic review articles were available covering studies up to 2017, searches were performed between January 1, 2018 to July 1, 2023 in the following databases: PubMed (MEDLINE), Science Direct, Scopus, and EMBASE databases using the following MeSH terms: ("Type I Diabetes Mellitus"[Mesh] OR "Insulin Dependent Diabetes Mellitus"[Mesh]) AND ("Hearing Loss"[Mesh]), where MeSH search was not available the following Boolean terms were used ("Type I Diabetes Mellitus" OR "Insulin Dependent Diabetes Mellitus") AND ("Hearing Loss").

### Study selection

All studies with confirmed diagnosis of T1D in adults and children as per World Health Organization (WHO) or American Diabetes Research Association (ADA) criteria with confirmed auditory evaluation using well-established measures such as PTA, ABRs and DPOAEs were included [46, 69–72]. Studies were excluded based on the following exclusion criteria: studies with no confirmed diagnosis of T1D and hearing loss, review articles, meta-analyses, abstracts only, conference proceedings, editorials/letters, case reports, or articles published before January 1, 2018. Additionally, animal models were excluded from analysis, as well as studies analyzing patients who had T2D or hearing loss due to a syndromic condition. All searched titles, abstracts, and full-text articles were independently reviewed by at least two trained reviewers (K.M., G.K., and R.M.). Disagreements regarding the criteria for including or excluding studies were resolved through mutual agreement among the reviewers, or by discussion with other investigators involved in this study. The initial screening of articles was conducted by examining their titles and abstracts, followed by a thorough full-text analysis.

### Data extraction

All data were extracted by at least two trained reviewers (K.M., G.K. and R.M.) and separated by age. Results were grouped together based on whether the study was performed in adults or children. The results were further separated based on the study’s objectives. Investigations evaluating the effect of diabetes on the auditory system were grouped together, and those assessing possible risk factors were also grouped together.

### Quality assessment

The Joanna Briggs Institute (JBI) Critical Appraisal Tools for Analytical cross-sectional studies and case control studies were used to perform quality assessment on eligible studies [73, 74]. For the cross-sectional studies, eight questions were evaluated: (1) were the criteria for inclusion in the sample clearly defined, (2) were the study subjects and the setting described in detail, (3) was the exposure measured in a valid and reliable way, (4) were objective/standard criteria used for measurement of the condition, (5) were confounding factors identified, (6) were strategies to deal with confounding factors stated, (7) were the outcomes measured in a valid and reliable way, and (8) was appropriate statistical analysis used. For the case-control studies, ten questions were evaluated: (1) were the groups comparable other than the presence of disease in cases or the absence of disease in controls, (2) were cases and controls matched appropriately, (3) were the same criteria used for identification of cases and controls, (4) was exposure measured in a standard/valid and reliable way, (5) was exposure measured in the same way for cases and controls, (6) were confounding factors identified, (7) were strategies to feal with confounding factors stated, (8) were outcomes assessed in a standard/valid and reliable way for cases and controls, (9) was the exposure period of interest long enough to be meaningful, (10) and was appropriate statistical analysis used. The questions were answered using “Yes”, “No”, and “Unclear”. At least two reviewers independently conducted this assessment (K.M., G.K., and R.M.), and any disagreements were resolved by consensus between the reviewers or discussion with other investigators of this study.

## Results

A total of 463 studies were retrieved using the predefined search algorithm as described in the methods section. After deduplication, 461 studies were included for title and abstract screening. After screening, 423 studies were excluded based on eligibility criteria and 38 articles were included for whole-text analysis. A total of 27 articles were then excluded as 3 had SNHL associated with a syndrome such as Wolfram, 19 did not include either SNHL or diabetes, 4 studied T2D instead of T1D, and 1 was performed on non-human subjects. Finally, 11 articles remained for inclusion in the literature review and qualitative analysis (Fig 1).

**Fig 2:**
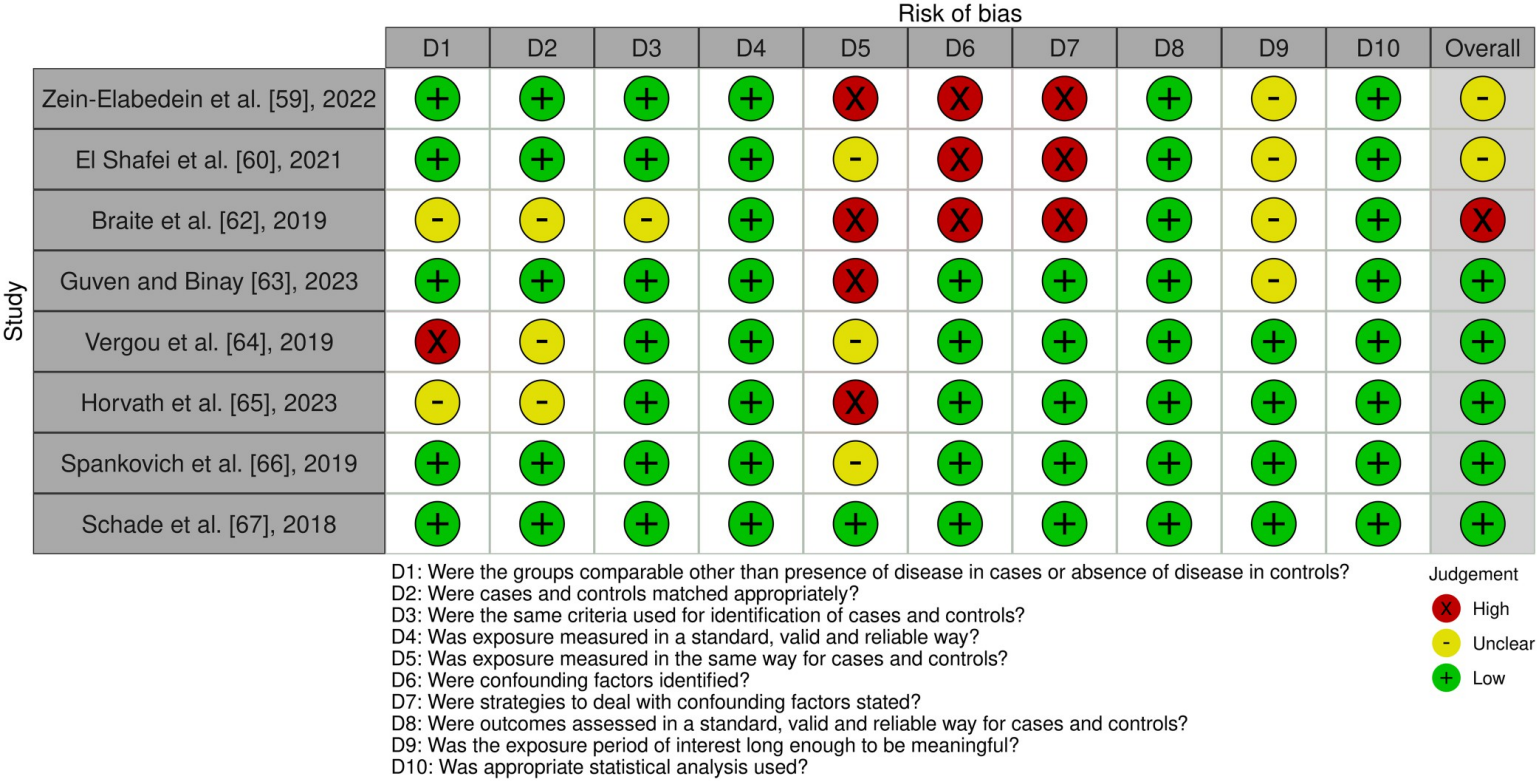
Assessment of risk of bias analysis of included case control studies using the Joanna Briggs Institute (JBI) critical appraisal tools.

**Fig 3:**
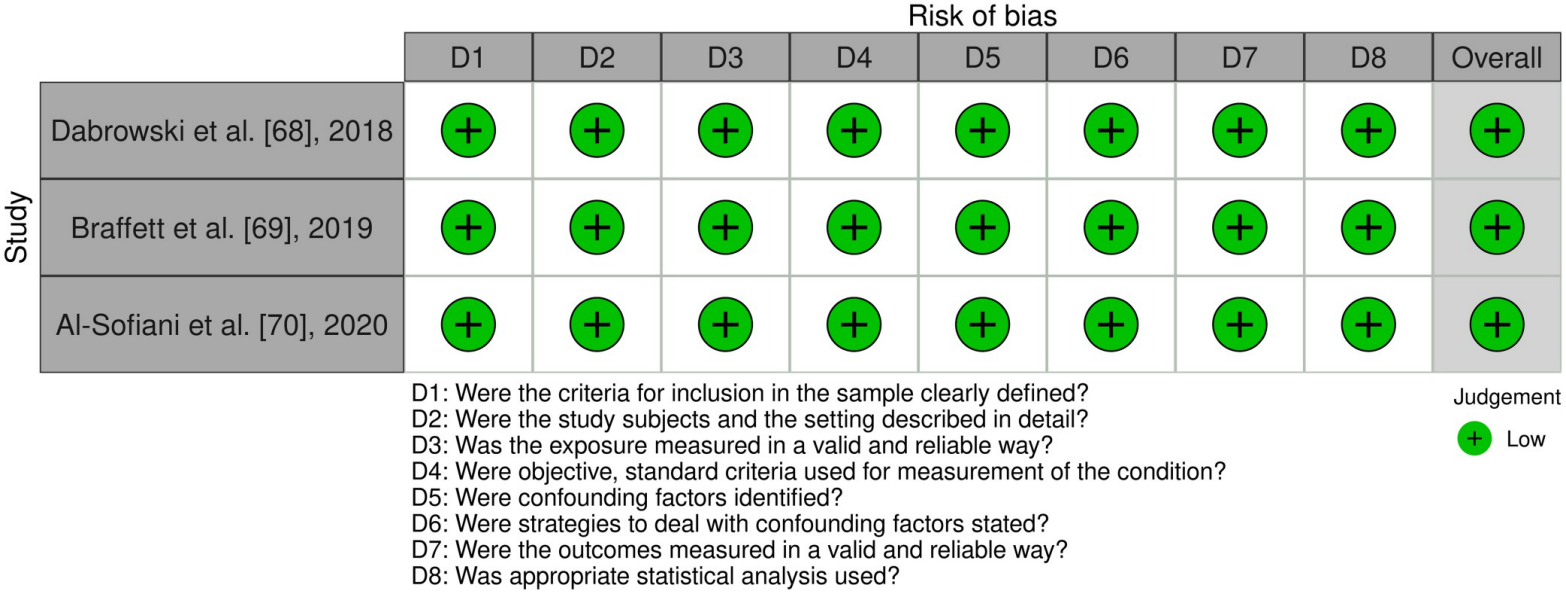
Assessment of risk of bias analysis of included cross sectional studies using the Joanna Briggs Institute (JBI) critical appraisal tools.

Eleven articles published between the years of 2018–2023 were included in this systematic review. A total of 5,792 patients were evaluated across the 11 articles included. Out of these studies, four investigations evaluated SNHL in children and four in adults whereas associated risks factors were evaluated in three studies. Most of the studies were case-control studies where the exposure group was patients with T1D, and the control group was patients without a diagnosis of diabetes mellitus. The risk of bias analysis for case control and cross-sectional studies is outlined in Figs 2 and 3. Eight of the eleven studies were found to have a low risk of bias, two a moderate risk of bias, and one a high risk of bias. Bias was mostly introduced in these studies due to the lack of appropriate testing in controls to rule out underlying undiagnosed T1D. Overall, the studies were determined to be of appropriate quality to be included in this systematic review. A summary of each study design, patient grouping, and results is presented in Table 1.

**Table 1:** A summary of main findings of the included studies.

Reference	Study	Population	Exposure	Comparison	Outcomes
Zein-Elabedein et al. [75], 2022	Case-control study	100 children aged 7–18	T1D	Non-diabetic controls	Significant difference in PTA at 8000 Hz SNHL in 14 diabetic children at 8000 Hz Positive correlation between disease duration and PTA threshold
El Shafei et al. [76], 2021	Case-control study	50 children aged 6–18	T1D	Non-diabetic controls	No difference in self-reported complaints of hearing loss or tinnitus No significant results regarding hearing loss
Braite et al. [78], 2019	Case-control study	101 children aged 7–18	T1D	Non-diabetic controls	Patients with T1D more likely to have self-reported hearing complaints Higher rate of altered contralateral acoustic reflex thresholds in T1D patients Significant differences in the MOC reflex inhibitory effect for individuals with T1D
Guven and Binay [79], 2023	Case-control study	42 children aged 5–18	T1D	Non-diabetic controls	Increased mean hearing threshold across every frequency, except 250 Hz, in T1D group Increased number of ears with a threshold above 15 dB for diabetic patients in the speech- and high-frequency ranges Subclinical hearing loss in T1D patients at EHF Associated with increased HbA1C
Vergou et al. [80], 2019	Case-control study	720 adults	T1D, T2D, and IFG	Non-diabetic controls	Impaired PTA and DPOAEs in the right ear of T1D patients Right ear results were similar between cases and controls
Horváth et al. [81], 2023	Case-control study	67 adults aged 19–60; split into 2 groups 19–39 and 40–60	T1D	Non-diabetic controls	For PTA, increased hearing impairment in T1D patients aged 40–60 Increased thresholds in T1D patients across both age groups Severely impaired OAEs at higher frequencies in T1D patients aged 40–60
Spankovich et al. [82], 2019	Case-control study	40 young adults aged 19–28	T1D	Non-diabetic controls	No significant difference in pure-tone thresholds or TEOAE Larger decrease in DPOAE fine structure peaks in T1D patients T1D may affect reflection source amplitude
Schade et al. [83], 2018	Case-control study	2,300 adults	T1D	Non-diabetic spouses of the participants	No difference in the odds of speech- or high-frequency hearing loss No difference in mean PTA Hearing impairment seemed to be correlated with increased HbA1C level
Dąbrowski et al. [84], 2018	Cross-sectional study	31 patients aged less than 45	T1D	None	Significant negative linear correlation between eGFR and hearing threshold Absence of TEOAE with significantly reduced eGFR Positive linear association between eGFR and ABR wave latency
Braffett et al. [85], 2019	Cross-Sectional Study	2,300 adults	T1D	None	Risk factors associated with hearing loss in diabetic patients included older age, male sex, less education, loud noise exposure, increased height/weight/waist circumference, hypertension, higher lipid levels, higher HbA1C, higher insulin dose, and presence of diabetic complications
Al-Sofiani et al. [86], 2020	Cross-Sectional Study	41 adults aged 20–60	T1D	None	19 of 30 adults with diabetes had high-frequency hearing loss Those with hearing loss were significantly older, had a significantly longer disease duration, and were significantly more likely to have peripheral neuropathy Those with hearing loss had significantly lower sRAGE

### Impact of T1D on hearing in children

Zein-Elabedein et al. conducted a case-control study in Egypt to understand how T1D affects cognitive function and hearing in children [75]. Cases included 50 children aged 7–18 with T1D. Children with other comorbid conditions or recent diabetic ketoacidosis were excluded. Controls included 50 non-diabetic children, matched for age, sex, and socioeconomic status. Those with a family history of diabetes mellitus or autoimmune disease were excluded. Additionally, the Stanford-Binet IQ test was administered, and children with a less than average normal score were excluded. Each study subject underwent a history and physical exam, as well as laboratory tests, including determination of HbA1C levels. Auditory evaluation included otoscopic exam, PTA, speech recognition threshold test using disyllabic Arabic words, and word discrimination test using phonetically balanced Arabic words. The researchers found that there was a statistically significant difference in the PTA between the groups at 8000 Hz (p = 0.001). The other frequencies were not significant. Additionally, 14 diabetic children were found to have bilateral SNHL at 8000 Hz. There was a correlation between duration of diabetic illness and PTA threshold. It was found that longer duration of illness was associated with higher PTA threshold, which is consistent with previous studies. However, the values were not significant. The speech reception thresholds were also not found to be significant, and all the children had a normal tympanogram [75].

El Shafei et al. also performed a randomized case control study to better understand how T1D affects the auditory system in children [76]. The study consisted of 25 children with T1D and 25 non-diabetic controls, all aged 6–18 years old. Children were excluded if they had known otologic disease or elevated blood sugar due to other conditions. All children underwent a full history, otologic exam, tympanometry and acoustic reflex testing, PTA, and speech audiometry. Results demonstrated there was no significant difference in self-reported complaints between the groups, including SNHL and tinnitus. Immitancemetry showed that, among cases, 23 patients had a type A tympanogram (normal middle ear, peak at a pressure of 0), 2 had a type C (significantly negative peak pressure, suggestive of eustachian tube dysfunction), and 4 had absent ipsilateral acoustic reflexes [77]. Among controls, 22 had a type A, 2 had a type B (flattened, indicative of pathology or fluid accumulation), 1 had a type C, and 2 had absent ipsilateral acoustic reflexes [77]. PTA showed that, among cases, 21 had normal hearing, 3 had mild bilateral high-frequency SNHL, and 1 had severe bilateral SNHL. Among controls, 22 had normal hearing, 1 had mild bilateral high-frequency SNHL, and 2 had bilateral conductive HL. This study did not observe statistically significant results regarding SNHL in individuals with T1D compared to the control group [76].

Subsequently, Braite et al. performed a study to investigate the effect of T1D on the auditory system in children and adolescents [78]. In this investigation, 50 of the participants had a diagnosis of T1D and 51 were controls. The participants were aged 7–18 years. Clinical data, including HbA1C, was obtained from medical records. Participants were also given a questionnaire to address any audiologic complaints. The hearing assessment included tonal audiometry, tympanometry, acoustic reflex, and DPOAE to evaluate the inhibitory effect of the medial olivocochlear reflex (MOC). The MOC inhibitory effect was considered present when the difference in amplitudes of DPOAEs was greater than zero. It was observed that participants with T1D were significantly more likely to complain of tinnitus, difficulties of speech/noise comprehension, difficulty understanding on the phone, difficulty understanding the television, and distraction in noise. PTA and tympanometry were similar between the groups. For the acoustic reflex, the T1D group had a higher rate of altered contralateral acoustic reflex thresholds at 4000 Hz (p = 0.0038) in the right ear and 1000 Hz (p = 0.036) in the left. Additionally, the acoustic reflex thresholds were higher in participants with T1D than controls. No significant difference was found between groups in amplitudes of DPOAEs. Finally, there was a significant difference in the MOC reflex inhibitory effect for individuals with T1D at 2000 Hz in the right ear and 6000 Hz in the left. In patients with diabetes, there was a higher number of absent MOC reflex at high frequencies. These findings suggest early dysfunction in the efferent auditory system in patients with T1D [78].

Guven and Binay performed a study to demonstrate the importance of extended high frequency (EHF) evaluation in children with T1D [79]. In this study, forty-two children aged 5–18 years were included in the study, 21 of which had T1D and 21 of which were non-diabetic controls. Children were excluded if they had known trauma, SNHL, otologic disease, or exposure to ototoxic drugs. Comprehensive pediatric and ENT exams were performed, and lab reports were obtained, including determining levels of fasting blood glucose, urea nitrogen, creatinine, cholesterol, triglycerides, LDL-C, HDL-C, and HbA1C. Audiological exam included PTA, tympanometry, and AR testing as well as evaluation of EHF (14,000, 16,000, and 18,000 Hz). The diabetic group had higher mean hearing thresholds at every frequency, except 250 Hz, and were statistically significant at 500, 2,000, 4,000, and 8,000 Hz. Additionally, the diabetic group had a significantly higher number of ears with a threshold above 15 dB at 8,000, 14,000, and 18,000 Hz. They also found significant results in the number of ears with a threshold above 15 dB for T1D patients in the speech-frequency (500–4,000 Hz) and high-frequency (4,000–8,000 Hz) ranges. Interestingly, it was observed that when the audiometry included EHF, it revealed subclinical hearing impairment in some study subjects, with a significantly higher prevalence in the diabetic group. Finally, they found that at 2,000 and 4,000 Hz, patients with thresholds above 15 dB had significantly higher HbA1C levels (p < 0.05). This study suggests that the use of EHF testing may allow for detection of subclinical SNHL in children with T1D. This will allow providers to closely monitor their glucose levels to prevent further damage and decline [79].

### Impact of T1D on hearing in adults

Vergou et al. performed a study to investigate the impact of glucose disorders on hearing function in adults [80]. In total, 499 patients were randomly selected to participate in the study; of those, 51 had a diagnosis of T1D, 188 had T2D, and 39 patients had impaired fasting glucose (IFG), defined as glucose greater than 100 mg/dL. These participants were matched with 221 non-diabetic controls. Exclusion criteria included ototoxic drug use in the previous three months, ear surgery or infection, significant noise-induced hearing loss, evidence of fluid behind the tympanic membrane (TM), flat tympanogram, Meniere’s disease, or labyrinthitis. Participants completed a questionnaire on their hearing-related history and demographics. Measurements were taken, including blood pressure, waist circumference, fasting blood glucose, creatinine, lipids, and HbA1C. The hearing exam included otoscopy, a tympanogram, pure-tone air and bone-conduction studies, and DPOAEs. The researchers found there was no significant difference in exposure to noise between the experimental groups and controls. Subjects with T1D had comparable PTA and DPOAEs in the left ear compared to controls. However, the right ear was significantly lower (35.3% vs. 56.9% and 13.7% vs. 24.2%, p < 0.001 and p = 0.044, respectively). Additionally, there was a correlation with age. T1D seemed to have a greater effect on younger patients who did not have the added effect of presbycusis and cumulative noise exposure. DM may intensify the effects of aging on the inner ear structures. The authors indicated that the DPOAE results suggest an impairment in the micromechanical property of the cochlea in patients with T1D. Previous studies had only addressed differences in PTA, which were not as significant. DPOAEs are a more objective and sensitive measure, which is a strength of this study. It was suggested that differences in study results are likely due to differences in glycemic control among participants [80].

Horváth et al. also conducted a prospective observational study to evaluate changes in the auditory system in patients with T1D [81]. In this study, forty-two patients with a diagnosis of T1D were included and were matched with 25 non-diabetic controls. They were divided into groups based on age (19–39 and 40–60 years). Patients were excluded if they were less than 18 years old, had occupational noise exposure, acute infection in the middle ear or upper airway, or known disease of the auditory system. Clinical data was collected, including c-peptide or antibody measurements, HbA1C levels, creatinine, estimated glomerular filtration rate (eGFR), and T1D-related complications, such as neuropathy, retinopathy, and nephropathy. Audiologic data included immittance audiometry, PTA, OAE, and ABR. For PTA, researchers did not find that there was hearing impairment in the 19–39 age group. However, hearing impairment was more common in the diabetic group compared to controls in the 40–60 age group. In the 19–39 age group, thresholds were higher in the diabetic group compared to controls for frequencies 500–4000 Hz on the right and 2000 Hz on the left. In the 40–60 age group, there was a significant difference between the groups at 4000 and 8000 Hz, with controls having normal hearing tests and diabetic patients having varying degrees of hearing loss. Both T1D and age had an effect on hearing. For OAE, values were relatively similar for the 19–39 group. There was only a moderate impairment found in the left ear of diabetic patients at 8000 Hz. Conversely, diabetic patients in the 40–60 group had severely impaired OAEs at higher frequencies compared to controls. ABRs were only conducted on diabetic patients. Possible retrocochlear lesions were identified in 15% of patients aged 19–39 and 25% of patients aged 40–60. However, this was not statistically significant (p = 0.4538). This demonstrates that T1D may damage the hearing system, and this effect may increase with age [81].

Additionally, Spankovich et al. performed a cross-sectional study to evaluate the effect that T1D has on the cochlea in young adults [82]. The study consisted of 20 patients with T1D and 20 matched non-diabetic controls. The participants were aged 18–28 years, had normal audiologic exams, normal otoscopic exams, were nonsmokers, and did not have exposure to ototoxic drugs. Their cochlear function was evaluated using behavioral pure-tone thresholds, transitory evoked otoacoustic emission (TEOAE), and DPOAE fine structure. The researchers found that there was no significant difference between the groups for behavioral pure-tone thresholds for frequencies 250 to 16000 Hz. There were also no statistically significant findings between the groups for TEOAE measurements. In terms of DPOAE fine structure, as decibel level increased, the number of fine structure peaks decreased. However, the decrease was significantly larger in the control group at 50 dB SPL (p < 0.05), indicating reduced cochlear function. T1D also seemed to have an effect on the reflection source amplitude at 35, 50, and 65 dB, but there was no difference found in the distortion source. The researchers speculated that the lack of significant findings across other measures was probably due to good glycemic control across the participants. It was suggested that DPOAE fine structure analysis may be a useful method to detect reduced cochlear function and cochlear pathology early in the diabetic disease course [82].

Finally, Schade et al. performed a study to determine the effects of T1D on hearing function [83]. This study utilized the same participants as used previously in the Diabetes Control and Complications Trial/Epidemiology of Diabetes Interventions and Complications (DCCT/EDIC). This study was a randomized controlled trial with 1,441 participants aged 13–39 years to compare intensive vs. conventional diabetes therapy and its effects on diabetic complications. This investigation utilized 1,150 surviving members from DCCT/EDIC trial to study their hearing function. Non-diabetic spouses of the participants were used as controls, under the assumption that they were matched on demographics, including age, sex, race, and socioeconomic status. Participants underwent standard audiological testing with PTA and completed a questionnaire about self-perceived hearing loss. The researchers found there was no significant difference in the odds of speech or high-frequency hearing impairment in either ear compared to controls. Additionally, there was no significant difference in mean PTA between the groups. These results were also found to be independent of HbA1C levels. The researchers further evaluated if there was a difference in hearing depending on which group the diabetic patients had participated in previously, intensive vs. conventional. They found that for every 10% increase in mean HbA1C, there was a 30% and 17% increase in speech and high-frequency SNHL, respectively. This demonstrates that long-term glycemia may be associated with hearing difficulty [83].

### Risk factors for SNHL in individuals with T1D

Dąbrowski et al. performed a study to investigate whether there is a correlation between hearing function and renal function in adults with T1D [84]. The cohort consisted of 31 patients, who had a diagnosis of T1D, 9 of whom were women. The participants were younger than 45 years and had a disease duration of less than 10 years. T2D and participants with significant noise exposure or ototoxic medication use were excluded. For each subject, BMI, blood pressure, lipid and creatinine level, HbA1C, and urinary albumin excretion (UAE) were measured followed by the calculation of eGFR. Audiologic assessment was also done on each patient, including PTA, TEOAE, and ABRs. Mild and moderate hearing impairment were determined to be thresholds exceeding 20 and 40 dB, respectively. Lack of otoacoustic emission was considered to be a mean TEOAE amplitude below 6 dB in a 1.2–3.5 kHz band range. The researchers observed mild to moderate bilateral SNHL in seven of the subjects using PTA. These patients had a significantly lower eGFR (108.8 vs. 121.7, p = 0.047), but were also significantly older (35.0 vs. 27.9, p = 0.016). Also, a significant negative linear correlation between eGFR and hearing threshold was found at 4, 6, 8, and 12 kHz using PTA. For TEOAE, absence of TEOAE was found in at least one ear for 7 patients. These patients had significantly lower eGFR (103.1 vs. 123.3, p < 0.001). No correlation was found between UAE and TEOAE. Finally, ABR found a significant positive linear association between eGFR and wave III latency and interval I-III. There was also a positive correlation between UAE and latency of wave III, wave V, and interval I-III. This study suggests a possible relationship between hearing and renal function in patients with T1D [84].

Additional risk factors were assessed by Braffett et al. by conducting a follow up study performed by Schade et al [85]. It was observed that speech-frequency SNHL was significantly associated with older age, male sex, less education, loud noise exposure, increased height/weight/waist circumference, hypertension, higher lipid levels, higher HbA1C, higher insulin dose, and presence of diabetic complications. Interestingly, light to moderate alcohol use and increased total cholesterol were associated with lower odds of speech-frequency SNHL. The same risk factors causing speech-frequency SNHL were also associated with high-frequency SNHL. However, risk factors also included nonprofessional/nontechnical occupations, increased systolic blood pressure, lower LDL cholesterol, and microalbuminuria. Furthermore, the presence of coronary artery calcifications and intima-media thickness (IMT) were significantly associated with high-frequency SNHL, but not speech-frequency. The association between IMT and high-frequency SNHL was only significant in males. This study helped to identify potential risk factors that can lead to SNHL in individuals with T1D [85].

Finally, Al-Sofiani et al. investigated the effects of duration of illness, HbA1C levels, and diabetic complications as well as select serum and urinary markers may have on SNHL in individuals with T1D [86]. The study included 30 subjects with T1D, aged 20–60 years, and 11 non-diabetic controls. The participants underwent PTA and neuropathy screening as well as blood and urine samples were obtained. Serum levels of C-reactive protein (CRP), vascular endothelial growth factor (VEGF), and soluble receptors for advanced glycation end-product (sRAGE) as well as urinary levels of isoprostane were measured. It was found that 19 of the 30 adults with T1D had high-frequency SNHL. Compared to the other participants with T1D that were found to have normal hearing, those with SNHL were significantly older, had a significantly longer disease duration, and were significantly more likely to have peripheral neuropathy. Individuals with T1D who were found to have high-frequency SNHL had significantly lower serum sRAGE. This is consistent with the theory that sRAGEs are protective against AGE toxicity and diabetic complications. There was no significant difference in CRP, VEGF, or urinary isoprostane [86].

## Discussion

The findings from the 11 articles reviewed suggest there may be a correlation between T1D and SNHL. However, the results were heterogenous, and further studies with large cohorts are warranted to confirm the prevalence of SNHL in individuals with T1D. The goal of this article is to bring attention to the possibility of SNHL in individuals with T1D, encouraging clinicians to consider auditory evaluations as part of the comprehensive care for these patients.

### Children

Three of the four studies demonstrated that children with T1D exhibit hearing impairment compared to non-diabetic controls. The children with T1D had significantly different PTAs at 8000 Hz, an increased mean hearing threshold, altered acoustic reflex thresholds, and problems with the MOC reflex inhibitory effect [75, 78, 79]. There was also a correlation between duration of diabetic illness and PTA threshold, as well as subclinical HL found at EHF. However, these results were not significant [75, 79]. Results were mixed in terms of self-reported symptoms. El Shafei et al. did not find a significant difference in self-reported tinnitus and HL, but Braite et al. did [76, 78]. There were no significant differences in DPOAEs [78]. Although some findings were mixed, these results suggest that T1D can result in hearing impairment, especially at higher frequencies.

### Adults

The four studies evaluated suggest that there are differences in the auditory system between patients with T1D and healthy controls, but many results were heterogenous. The two studies found there was a significant difference between PTAs [80, 81], but one found there was no significant difference [83]. Additionally, one study found there was a significant difference in thresholds between the groups [81], but another study did not [82]. However, results showed that PTAs and OAEs were significantly worse in older diabetic individuals compared to younger subjects, indicating an association with age [81]. Also, high levels of HbA1C correlated with an increase in speech and high-frequency SNHL [83]. Finally, DPOAE fine structure showed a larger decrease with increasing decibel level in diabetic patients compared to controls, which may be an early marker of cochlear dysfunction [82]. These results suggest T1D may impair the auditory system, and this correlation is exacerbated by age and increased HbA1C levels.

### Risk factors

Studies found there were several risk factors associated with SNHL in individuals with T1D. In general, SNHL was associated with older age, male sex, less education, loud noise exposure, increased height/weight/waist circumference, hypertension, higher lipid levels, higher HbA1C, higher insulin dose, longer disease duration, and presence of diabetic complications [85, 86]. Dąbrowski et al. also found an association between SNHL and eGFR, demonstrating that decreased eGFR is associated with increased hearing threshold and absent TEOAE [84]. These findings suggest microvascular damage affecting both the kidney and the inner ear. Many of these risk factors indicate that aggressive glycemic control may be able to limit the damage to the auditory system.

## Limitations

There are a few limitations of this systematic review article. Many of the included studies utilized small and varied sample sizes as well as had a retrospective design. This presents a significant limitation in the field, as the limited number of participants and the diversity in study designs and populations make it challenging to draw generalizable conclusions. The small sample sizes reduce the statistical power of these studies, potentially leading to less reliable or inconclusive results. Additionally, the studies did not test controls to rule out undiagnosed pre-diabetes or diabetes mellitus. Although it is unlikely that a large number of control participants had undiagnosed T1D, performing glucose tests on this group could have strengthened the methods and decreased bias. Finally, the children included had a short duration of diabetes, so it is possible they will encounter complications in the future that were not present at the time of the study. Furthermore, a comprehensive battery of sensitive hearing tests was not employed to detect SNHL especially at subclinical levels.

The other limitation of our article is that it does not distinctly specify the gender more susceptible to the impact, nor does it provide clarity regarding the professions of those affected. One of the greatest issues is that SNHL in individuals with T1D is an emerging research area. Most of the published studies might not have had access to detailed demographic or occupational data. This can be attributed to reliability on secondary data sources, which may not include comprehensive information about participants’ gender or professions. It may be possible that in some studies information might have been collected regarding gender and profession, but the sample size or the results might not have been statistically significant to draw reliable conclusions. Hence, these details were not emphasized in the article.

Furthermore, the heterogeneous outcomes derived from only 11 articles included in this systematic review may not sufficiently establish a direct correlation between T1D and SNHL. The current data, while suggestive, highlights the need for more extensive, well-designed studies to conclusively establish the relationship between T1D and SNHL. Longitudinal studies would be valuable in understanding progression and causal relationships.

Besides these limitations, the studies searchable through PubMed (MEDLINE), Science Direct, Web of Science, Scopus, and EMBASE databases and mapped to the MeSH search terms were included in this systematic review, which may have impacted the results reported.

## Conclusion and future directions

The results of this systematic review suggest that T1D may be correlated with SNHL. This is evidenced by abnormal audiological outcomes compared to the healthy controls. However, a few studies did not observe statistically significant differences in hearing thresholds between individuals with T1D and control group. It is possible that SNHL is prevalent in T1D group but at subclinical levels, therefore, more sensitive hearing tests are warranted for its detection. In addition, there is a need to longitudinally follow T1D individuals over an extended period of time as they may develop SNHL during the advanced stages of disease. Overall, the findings from PTA, ABR, TEOAE, and DPOAE measures suggest that T1D affects the auditory system at multiple levels including damage to OHCs, SV and SL, which may lead to hearing impairment. These results are in agreement with data from temporal bone studies of T1D individuals demonstrating loss of OHCs, atrophy of SV, and loss of SL cells [19, 87, 88]. In addition, there was significant thickening of the walls of vessels in the basement membrane and in the SV. It was suggested that microangiopathy associated with T1D adversely affects the vasculature in the inner ear, damaging cochlear capillaries that lead to atrophy of SV, and subsequently loss of OHCs, resulting in hearing impairment.

As T1D is an autoimmune disease, it is possible that there is a discernible connection between autoimmunity and the occurrence of SNHL. In autoimmune conditions such as T1D, the body produces autoantibodies that could theoretically cross-react with tissues in the inner ear. However, it is important to note that the direct involvement of autoantibodies in causing SNHL in individuals with T1D is not well-established and would warrant further studies for definitive conclusions. Most research on T1D and hearing impairment has focused on broader metabolic and vascular effects rather than specific autoimmune mechanisms. Future investigations in this area could significantly enhance our understanding of the intersection between autoimmune disorders and hearing health.

There is emerging research suggesting that COVID-19 might act as a trigger for the onset or acceleration of T1D in some individuals, particularly those with a genetic predisposition [89–92]. The virus may induce stress on the pancreatic beta cells or trigger an autoimmune response. Individuals with T1D might experience a cumulative effect of the disease and COVID-19, leading to an increased risk of complications, including hearing impairment. In addition, COVID-19 is known to cause systemic inflammation and has been associated with a range of complications, including effects on endocrine function [93–96]. This could potentially impact the progression of pre-existing autoimmune diseases such as T1D. The systemic inflammatory response from COVID-19 might contribute to conditions that could exacerbate hearing problems, especially in individuals with pre-existing conditions such as diabetes. Given the novelty of COVID-19 and its myriad impacts on health, ongoing research is essential to understand its full implications, particularly concerning chronic diseases such as T1D. The intersection of COVID-19 with chronic diseases such as T1D and their complications, including auditory function and hearing impairment, is an important area for future investigations.

SNHL in T1D is further exaggerated in older patients as well as individuals with a longer disease duration and poorer glycemic control. These findings suggest that tight glycemic control may help to limit damage to the auditory system and hence preventing SNHL. Healthcare providers should be aware of this correlation as they counsel patients on the importance of tight glycemic control. Additionally, it may be beneficial to include regular hearing tests for diabetic patients, similar to the recommended renal and ophthalmological screenings. This would allow providers to intervene early in the disease course leading to better clinical outcomes. There is also a need to understand the precise molecular mechanisms underlying SNHL in T1D which will provide novel avenues for its effective management leading to improved quality of life of affected individuals and their families/caregivers.

## Supporting information

S1 Checklist PRISMA 2020 checklist. (PDF)

## Abbreviations

ABRs: Auditory brainstem responses
AEP: Auditory evoked potential
DPOAEs: Distortion product otoacoustic emissions
EHF: Extended high frequency
MOC: Medial olivocochlear
OC: Organ of Corti
OHCs: Outer hair cells
PTA: Pure tone average
SNHL: Sensorineural hearing loss
SV: Stria vascularis
T1D: Type 1 diabetes
T2D: Type 2 diabetes
TEOAEs: Transitory evoked otoacoustic emissions
